# Severe rheumatic mitral stenosis and ARDS in pregnancy managed with percutaneous mitral commissurotomy and ECMO: a case report and literature review

**DOI:** 10.1186/s12884-026-09013-4

**Published:** 2026-04-08

**Authors:** Carlos Enrique Vesga-Reyes, Paula Andrea Cárdenas-Marín, Maria Juliana Reyes-Cardona, Pastor Olaya, Jorge Alexander Zambrano-Franco, Jairo Sanchez-Blanco, Miller Giraldo-Sandoval, Diego Fernando Bautista-Rincon, Camilo Andres Calderon-Miranda

**Affiliations:** 1 Departamento de Cardiología, Fundación Valle del Lili, Carrera 98 No. 18 - 49, Cali, 760032 Colombia; 2 Facultad de Ciencias de la Salud, Universidad Icesi, Calle 18 No. 122-135, Cali, 760031 Colombia; 3 Departamento de Medicina Interna, Fundación Valle del Lili, Carrera 98 No. 18 - 49, Cali, 760032 Colombia; 4 Unidad de Intervencionismo Vascular, Fundación Valle del Lili, Carrera 98 No. 18 - 49, Cali, 760032 Colombia; 5 Departamento de Medicina Crítica y Cuidado Intensivo, Fundación Valle del Lili, Carrera 98 No. 18 - 49, Cali, 760032 Colombia

**Keywords:** Mitral valve stenosis, Heart valve diseases, Rheumatic heart disease, Pregnancy complications, Cardiovascular, Extracorporeal membrane oxygenation, Balloon valvuloplasty, Echocardiography, Transesophageal, Respiratory distress syndrome, Adult

## Abstract

**Background:**

Rheumatic mitral stenosis (MS) remains a leading cause of cardiovascular complications in pregnancy, particularly in low-resource settings. Hemodynamic changes during pregnancy can precipitate decompensation in previously asymptomatic patients, increasing the risk of maternal and fetal morbidity. Early recognition and management, including percutaneous mitral commissurotomy (PMC) in selected cases, are crucial to optimizing outcomes.

**Case presentation:**

We present the case of a 32-year-old pregnant woman (G4P3) at 22.6 weeks of gestation who developed severe respiratory distress and cardiogenic shock due to previously undiagnosed severe rheumatic MS. Initial management of respiratory distress at a rural hospital included inhaled beta-agonists; however, her condition rapidly deteriorated, requiring orotracheal intubation and urgent transfer to a tertiary care center. Transthoracic echocardiography confirmed severe MS (mitral valve area: 1.3 cm², mean gradient: 17 mmHg) with a severely dilated left atrium. A respiratory molecular panel was positive for Influenza A. Despite medical therapy, she developed refractory hypoxemia, distributive and cardiogenic shock, necessitating escalating vasopressor support and mechanical ventilation. A multidisciplinary team decision led to urgent PMC, successfully performed with a post-procedure mitral valve area of 2.6 cm². However, due to persistent respiratory failure, veno-venous extracorporeal membrane oxygenation (ECMO) was initiated, with successful decannulation after 33 days. The patient recovered without residual respiratory distress, and subsequent obstetric ultrasounds confirmed fetal viability and normal growth. She later underwent an uneventful delivery at 38 weeks, with favorable maternal and neonatal outcomes.

**Conclusions:**

This case underscores the importance of early diagnosis, echocardiographic assessment, and multidisciplinary management in pregnant patients with severe MS. PMC remains the preferred intervention for severe symptomatic MS during pregnancy, significantly improving hemodynamics and reducing maternal risk. In cases of severe decompensation, ECMO serves as a life-saving bridge to recovery, ensuring both maternal stabilization and favorable perinatal outcomes. This case report highlights the need for preconception counseling, early intervention, and individualized care in high-risk pregnancies complicated by valvular heart disease.

**Supplementary Information:**

The online version contains supplementary material available at 10.1186/s12884-026-09013-4.

## Background

In pregnancy, mitral valve stenosis (MS) is recognized as the most common valvular disease, accounting for up to 56–89% or cardiac diseases during pregnancy in developing countries compared to a prevalence of 1–2% in developed countries [1].

MS is diagnosed when the mitral valve area (MVA) is ≤ 1.5 cm² and is more frequently observed in women. Rheumatic fever remains the leading cause of mitral stenosis (MS). Women with severe MS often struggle to handle the hemodynamic and cardiovascular demands of pregnancy leading to exacerbation of their condition. MS is associated with increased risk of maternal and fetal morbidity [2–4].

This case highlights important considerations for the management of significant valvular heart disease, with percutaneous mitral commissurotomy (PMC), as well as the role of extracorporeal membrane oxygenation (ECMO) in a patient with concomitant severe MS and acute respiratory distress syndrome (ARDS).

## Case presentation

A 32-year-old pregnant woman (G4P3) at 22.6 weeks of gestation, with no known prior medical history, presented to a rural hospital with flu-like symptoms and dyspnea. She was discharged with inhaled beta-agonist therapy but returned with respiratory distress, a heart rate of 122 beats per minute, an arterial oxygen saturation (SaO₂) of 75% despite fraction of inspired oxygen (FiO₂) 100% and a blood pressure of 142/84 mmHg. Given the severity of her condition, she required orotracheal intubation and was emergently transferred to a higher-complexity center.

Upon admission, bedside ultrasonography revealed abundant B-lines and hepatization of the left lung. A respiratory panel tested positive for Influenza A. Obstetric ultrasound confirmed a single live fetus with no evidence of malformations, growing within normal percentiles.

Transthoracic echocardiography (TTE) revealed a preserved left ventricular ejection fraction of 60% and a severely dilated left atrium (area of 31 cm²). The MV exhibited diffuse thickening of both leaflets, commissural fusion, and minimal calcification, consistent with rheumatic valve disease leading to severe stenosis and mild regurgitation. MS parameters included a maximum velocity of 2.8 m/s, a peak gradient of 31 mmHg, mean gradient of 17 mmHg and MVA of 1.3 cm² by planimetry.

The patient was admitted to intensive care due to hypoxemic respiratory failure, cardiogenic and distributive shock requiring escalating dual vasopressor support with norepinephrine and vasopressin. She had refractory hypoxemia (PaFi < 100) under invasive mechanical ventilation, sedation, neuromuscular blockade, and failed pronation cycle. Bloodwork showed a pH 7.17, pCO2 67 mmHg, pO2 83 mmHg and PaO2/FiO2 83 mmHg, chest radiograph with bilateral perihilar infiltrates. Transpulmonary thermodilution presented with an elevated extravascular lung water index (37 mL/kg), as well as an elevated pulmonary vascular permeability index (7.2), consistent with a non-cardiogenic pulmonary edema, along with normal filling pressures, expected in ARDS. A multidisciplinary team involving clinical cardiology, critical care, cardiovascular surgery and interventional cardiology specialists determined that PMC was the best course of action.

**Fig. 1:**
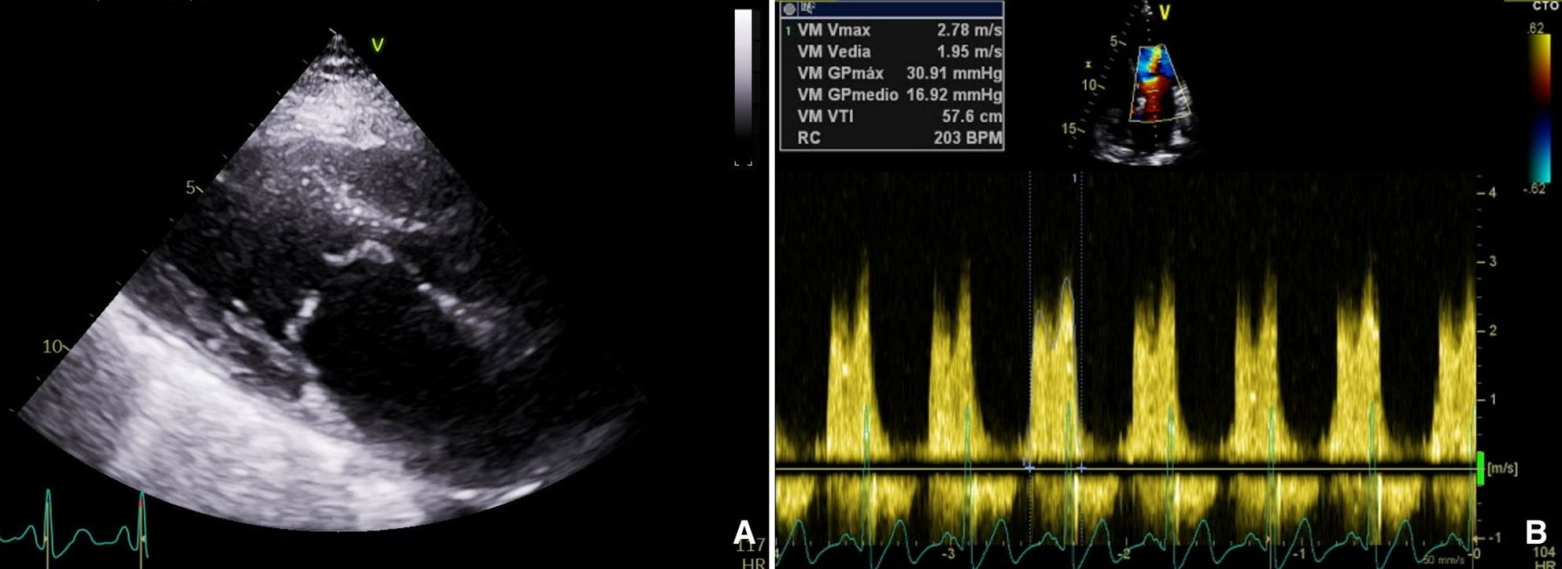
Transthoracic echocardiogram. **A** Parasternal long-axis view. Mitral valve with diffuse thickening of both leaflets, restriction in their marginal opening with bicommissural fusion, minimal calcification. **B** Spectral Doppler. Mitral stenosis with a maximum velocity of 2.8 m/s, a peak gradient of 31 mmHg and mean gradient of 17 mmHg. Mild mitral regurgitation

A pre-procedural transesophageal echocardiogram (TEE) confirmed severe MS with a mean gradient of 17 mmHg and a MVA of 1.3 cm² by planimetry (Wilkins score: 6 points). Additionally, rheumatic aortic valve involvement was observed, with mild regurgitation (Fig. 1) (Supplementary file Video 1).

**Fig. 2:**
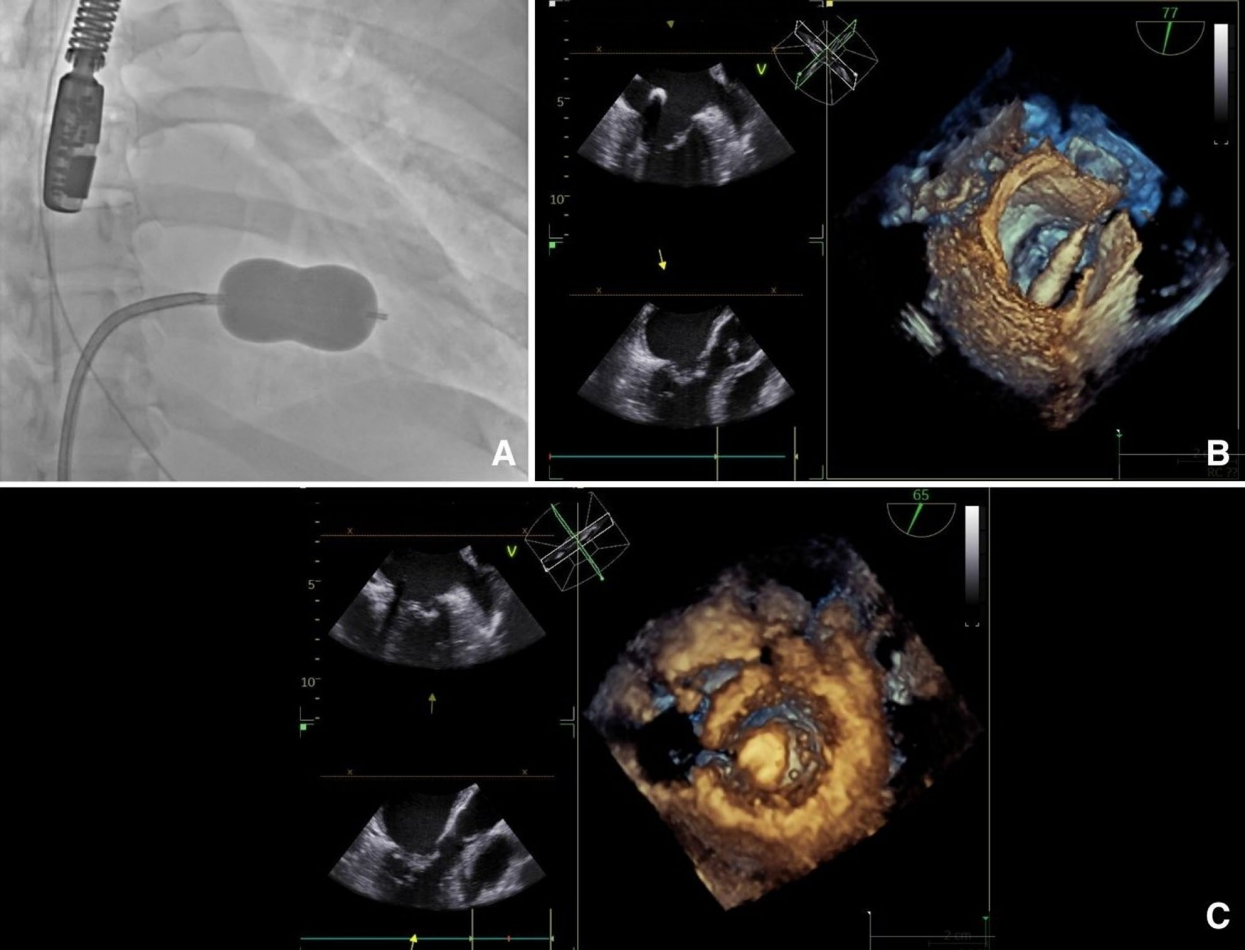
Percutaneous mitral commissurotomy. **A** Fluoroscopy showing inflation of a properly positioned valvuloplasty balloon. **B** Transesophageal echocardiogram (TEE), 77°. Advancement of balloon valvuloplasty catheter. **C** TEE, 65°. The balloon is inflated, opening the mitral leaflets, separation post balloon inflation is shown here

**Fig. 3:**
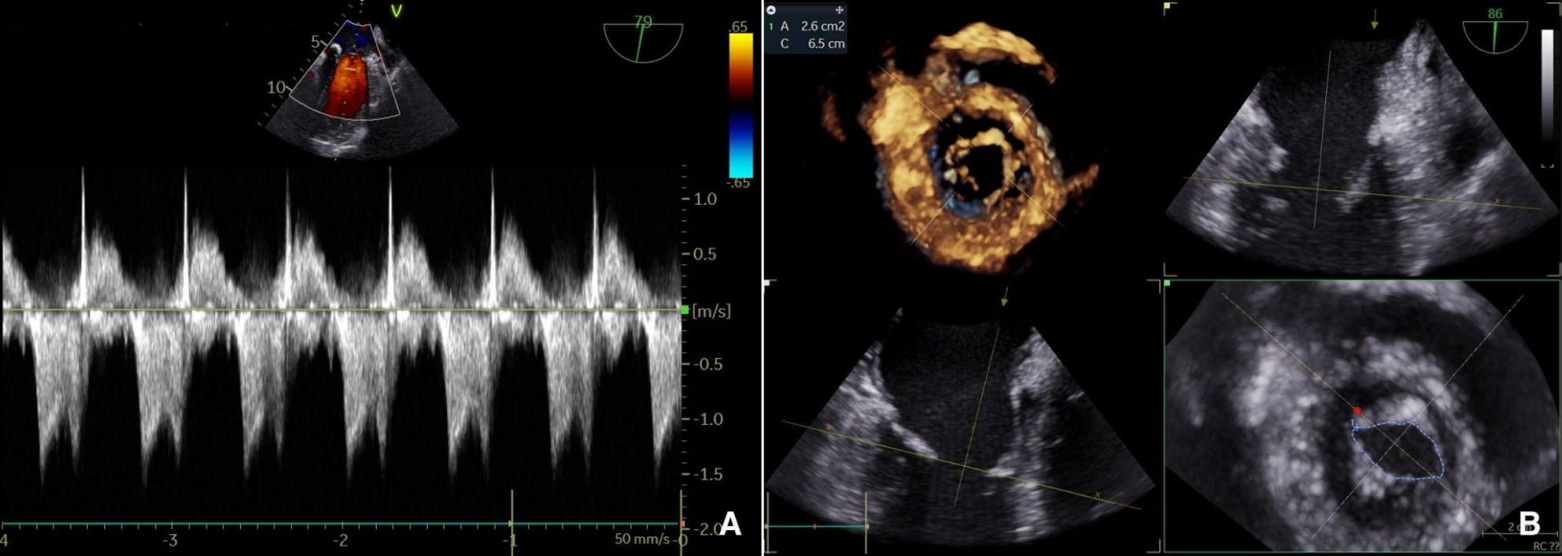
Post-procedural transesophageal echocardiogram. Significant improvement in mitral valve opening. **A** Maximum velocity: 1.5 m/s, peak gradient: 9 mmHg, mean gradient: 3.8 mmHg. **B** Mitral valve area by 3D planimetry: 2.6 cm²

PMC was successfully performed under TEE guidance. An 8 Fr femoral venous introducer was placed, followed by transseptal puncture using a BRK1 guidewire. A Protak guidewire was positioned in the left atrium, and an Inoue No. 28 balloon was advanced into the left ventricle for mitral valvuloplasty, which was completed without complications (Fig. 2) (Supplementary file Video 2, balloon inflation and commissural splitting). Post-procedural echocardiography demonstrated marked improvement in MV opening, with a MVA of 2.6 cm² by 3D planimetry, maximum velocity of 1.5 m/s, peak gradient of 9 mmHg, mean gradient of 3.8 mmHg, and trivial residual regurgitation (Fig. 3).

After a successful valvuloplasty, the patient presented with a deterioration of ventilatory mechanics given by a descent of compliance to 15 cmH_2_O/mL and non reclutability with a recruitability index of 0.2 [5]. Due to ARDS with persistent hypoxemia, worsening ventilatory mechanics and a failed prone positioning attempt, veno-venous extracorporeal membrane oxygenation (ECMO) was initiated without complications and maintained for 33 days. Following successful decannulation, the patient transitioned to high-flow nasal cannula support for 20 days. Serial obstetric ultrasounds confirmed a viable fetus with normal growth parameters. She was ultimately discharged in stable condition, without oxygen supplementation, respiratory distress or peripheral edema.

She was later readmitted for contractions at 38 weeks and underwent vaginal delivery with favorable maternal-fetal outcomes. The newborn weighed 3160 gr and breathed spontaneously. Postpartum maternal clinical course was satisfactory without bleeding or cardiac symptoms, a subdermal implant was placed for contraception and she was discharged with her newborn two days after delivery. Long-term maternal cardiac function and valve durability could not be assessed, as follow-up occurred outside our institution.

## Discussion

Rheumatic heart disease (RHD) is the leading pre-existing cardiovascular disease in pregnancy in low-income countries, accounting for 56–89% of cardiac diseases [6]. RHD is a chronic condition resulting from damage to the cardiac valves due to acute rheumatic fever, an autoimmune reaction following pharyngitis by Streptococcus pyogenes, resulting from molecular mimicry between bacterial antigens and cardiac “M-protein”. The MV is involved in > 95% of cases of RHD, causing mitral regurgitation early in the course of the disease (frequently seen in children and young adults) and MS later on, presenting in older patients with a long asymptomatic period followed by gradual dyspnea on exertion and findings of right heart failure and pulmonary hypertension (PH) [7]. Aortic valve involvement is detected in 20–30% of cases, most frequently with concomitant MV disease. Approximately two thirds of patients with RHD are female, many of them first presenting during pregnancy as stenotic left valvular disease is poorly tolerated [8], with a high risk of adverse fetal-maternal outcomes in limited-resources settings.

Mitral RHD pathology is characterized by thickening of the leaflets, nodularity and commissural fusion, resulting in systolic and diastolic leaflet motion restriction and MV narrowing; sometimes exacerbated by chordal fusion and retraction. While the normal MVA is 4–6 cm², clinically significant MS is defined by a MVA ≤ 1.5 cm² and most patients are symptomatic when the MVA is less than 1 cm² [9].

Patients with MS most commonly present with dyspnea on exertion or related to conditions causing tachycardia, increased flow through the valve or lack of atrial contraction (e.g. Atrial fibrillation (AF)), which are associated with a rise of mean left-atrial pressure. Less frequent symptoms include hemoptysis, orthopnea, fatigue, palpitations due to AF and hoarseness due to left atrial enlargement. Complications include PH, right heart failure and thromboembolic events related to atrial dilation and AF [2, 3, 7, 9].

Pregnancy can precipitate symptoms related to previously asymptomatic MS due to increased intravascular volume and heart rate, especially in second and third trimesters, as the stenotic valve limits the capacity to increase cardiac output leading to retrograde congestion and insufficient uteroplacental blood flow. Our undiagnosed, previously asymptomatic patient presented with respiratory failure in the second trimester of pregnancy due to MS and concomitant Influenza A viral pneumonia.

Echocardiography is the imaging modality of choice for the diagnosis, classification and anatomical characterization of MS in pregnant and non-pregnant patients. The MVA measured by 2D planimetry serves as the reference standard for assessing MS severity, while the mean transvalvular gradient and pulmonary pressures indicate its hemodynamic impact and prognostic significance. Three-dimensional (3D) TTE planimetry may provide further diagnostic value. In most cases, TTE provides a satisfactory assessment. However, a TEE may be needed for a detailed anatomical evaluation before procedures or to exclude left atrial thrombus prior to PMC, for which echocardiography plays a relevant periprocedural role [3]. In the present case, TTE was used for the diagnosis of severe MS and detection of morphological abnormalities suggestive of a rheumatic etiology. TEE was used for preprocedural planning and intraprocedural guidance of PMC.

An increased risk of maternal and fetal complications has been reported in patients with MS. Prematurity occurs in 20–30% of cases, intrauterine growth restriction in 5–20%, and fetal mortality ranges from 1 to 5% [6, 10]. In the study by van-Hagen et al. [11], including 273 pregnant women with rheumatic MS, maternal death during pregnancy occurred in 1 patient (1.9% of severe MS) and 2 women died in the first 6 months postpartum. 23.1% required hospital admission for a cardiac cause. The main reason was heart failure, especially in patients with severe MS (49.1%). An intervention during pregnancy was performed in 5.9% of patients, 93% had PMC and the remaining had surgical valve replacement, all of them with favorable outcomes. Women with severe MS had an earlier delivery and newborns with lower birth weight.

Considering the high maternal and fetal morbidity related to MS, management of pregnant patients with MS must be guided by a multidisciplinary team involving cardiology and maternal-fetal medicine. Patients with significant MS should receive preconception counseling advising against pregnancy. Whenever possible, intervention -preferably percutaneous- should be considered before conception, even in asymptomatic women, especially if the MVA is <1 cm² [1, 6, 10].

However, often the onset of symptoms and diagnosis of MS take place during pregnancy, as occurred in our case. In this scenario, medical management includes selective beta-blockers, activity restriction and diuretics, avoiding volume depletion to preserve uteroplacental perfusion. Anticoagulation is indicated if AF, left atrial thrombosis or prior embolism are present [6, 10].

PMC should be considered in women with NYHA class III/IV or systolic pulmonary artery pressure ≥ 50 mmHg despite medical management [6, 10], in patients with favorable criteria according to the Wilkins, Cormier, and Echo scores, and who have no contraindications for the procedure. It is preferably performed after 20 weeks of gestation to avoid radiation exposure to the fetus in the first trimester of pregnancy. In a systematic review and meta-analysis of observational studies of PMC during pregnancy [12], the procedure was successful in 93.6% of cases with failure more often in patients with subvalvular disease. Maternal mortality was 6.5% in women who had a successful procedure and 31.6% in those whose procedure was unsuccessful. The most frequent complications were mitral regurgitation (12.7%) and restenosis (2.4%). Among neonatal complications the most common was low birthweight (5.4%), which may be attributed to the underlying disease rather than the procedure itself.

ECMO is a life-saving intervention used in pregnant patients with severe MS who develop refractory cardiogenic shock, pulmonary edema or severe respiratory failure despite optimal medical therapy and interventions [13, 14]. Our patient presented with persistent hypoxemic respiratory failure requiring prolonged ECMO and high-flow nasal cannula support with successful weaning.

Vaginal delivery is indicated in patients in NYHA class I/II without PH, while caesarean section is usually recommended in patients in NYHA class III/IV or with PH without possibility of prior intervention [6, 10]. Our patient was asymptomatic after discharge and underwent vaginal delivery with early favorable maternal and fetal outcomes. Long-term maternal follow-up beyond the postpartum period was not available, as the patient’s clinical care did not continue at our institution after hospital discharge.

## Conclusions

Rheumatic MS remains a significant contributor to maternal morbidity and mortality, particularly in resource-limited settings where RHD is prevalent. Pregnancy exacerbates the hemodynamic burden of MS, often leading to decompensation, heart failure, and increased fetal risks. Early diagnosis, close clinical and echocardiographic follow-up, and a multidisciplinary approach are essential for optimal maternal-fetal outcomes.

PMC remains the preferred intervention for symptomatic severe MS during pregnancy, offering substantial hemodynamic improvement while minimizing procedural risk. However, in cases of refractory respiratory failure or cardiogenic shock, ECMO may be required as a life-saving measure, as demonstrated in this case.

This report highlights and illustrates the importance of preconception counseling, early intervention, and individualized management in pregnant patients with MS, ensuring both maternal stabilization and favorable perinatal outcomes. While not generalizable, this case highlights key principles in the management of valvular heart disease during pregnancy.

## Supplementary Information

Supplementary Material 1: Video 1. Pre-procedural echocardiogram. Rheumatic mitral valve disease, with diffuse thickening of both leaflets, commissural fusion, little calcification, subvalvular thickening of the chordae tendineae. A. Transthoracic echocardiogram. Parasternal long-axis view. B. Transesophageal echocardiogram, 41°, focused on the mitral valve with limitation for valvular opening during systole and valvular thickening.

Supplementary Material 2: Video 2. Percutaneous mitral commissurotomy. Intraprocedural Fluoroscopy (A) and transesophageal echocardiography (B) guidance showing inflation of a properly positioned valvuloplasty Inoue No. 28 balloon.

## Data Availability

No datasets were generated or analysed during the current study.

## Abbreviations

AF: Atrial Fibrillation
ECMO: Extracorporeal Membrane Oxygenation
FiO₂: Fraction of Inspired Oxygen
G4P3: Gravida 4, Para 3
MVA: Mitral Valve Area
MS: Mitral Stenosis
MV: Mitral Valve
NYHA: New York Heart Association
PaFi: PaO₂/FiO₂ Ratio
PH: Pulmonary Hypertension
PMC: Percutaneous Mitral Commissurotomy
RHD: Rheumatic Heart Disease
SaO₂: Arterial Oxygen Saturation
TEE: Transesophageal Echocardiography
TTE: Transthoracic Echocardiography
VHD: Valvular Heart Disease
